# The Dopamine D4 Receptor Regulates Gonadotropin-Releasing Hormone Neuron Excitability in Male Mice

**DOI:** 10.1523/ENEURO.0461-21.2022

**Published:** 2022-03-02

**Authors:** Leigh Dairaghi, Stephanie Constantin, Andrew Oh, David Shostak, Susan Wray

**Affiliations:** Cellular and Developmental Neurobiology Section, National Institute of Neurological Disorders and Stroke/National Institutes of Health, Bethesda, MD 20892

**Keywords:** calcium imaging, dopamine, fertility, GnRH, patch clamp

## Abstract

Gonadotropin-releasing hormone (GnRH)-secreting neurons control fertility. The release of GnRH peptide regulates the synthesis and release of both luteinizing hormone (LH) and Follicle stimulation hormone (FSH) from the anterior pituitary. While it is known that dopamine regulates GnRH neurons, the specific dopamine receptor subtype(s) involved remain unclear. Previous studies in adult rodents have reported juxtaposition of fibers containing tyrosine hydroxylase (TH), a marker of catecholaminergic cells, onto GnRH neurons and that exogenous dopamine inhibits GnRH neurons postsynaptically through dopamine D1-like and/or D2-like receptors. Our microarray data from GnRH neurons revealed a high level of *Drd4* transcripts [i.e., dopamine D4 receptor (D4R)]. Single-cell RT-PCR and immunocytochemistry confirmed GnRH cells express the *Drd4* transcript and protein, respectively. Calcium imaging identified changes in GnRH neuronal activity during application of subtype-specific dopamine receptor agonists and antagonists when GABAergic and glutamatergic transmission was blocked. Dopamine, dopamine with D1/5R-specific or D2/3R-specific antagonists or D4R-specific agonists decreased the frequency of calcium oscillations. In contrast, D1/5R-specific agonists increased the frequency of calcium oscillations. The D4R-mediated inhibition was dependent on G_αi/o_ protein coupling, while the D1/5R-mediated excitation required G_αs_ protein coupling. Together, these results indicate that D4R plays an important role in the dopaminergic inhibition of GnRH neurons.

## Significance Statement

The convergence of information on neurons secreting gonadotropin-releasing hormone (GnRH) shape their secretory profile and consequently, fertility. Dopamine inhibits GnRH neurons, yet the specific dopamine receptor subtype(s) involved remain unclear. Using single RT-PCR, immunofluorescence, and dopamine receptor-specific pharmacological tools, we show here that dopamine D4 receptor (D4R) plays a role in the dopaminergic inhibition of GnRH neurons, adding a new tool for the modulation of reproductive function.

## Introduction

Dopamine is a regulator of reproductive function at many levels along the hypothalamic-pituitary-gonadal axis. A female-specific dopaminergic inhibitory tone, observed in developing rats (Lacau de Mengido et al., 1987; Becú-Villalobos and Libertun, 1995), influences the timing of puberty (Ruf and Holmes, 1974; Lamberts and Wuttke, 1981; Gerber et al., 1984; de Mengido et al., 1989). In seasonal breeders, dopamine is a component of anestrus (Lehman et al., 1996; Saxena et al., 2015). Ovarian dopamine receptors regulate ovulation (Venegas-Meneses et al., 2015) and dopaminergic neurons control anterior pituitary function (Hnasko et al., 2007; Gonzalez-Iglesias et al., 2008). In the hypothalamus, tyrosine hydroxylase (TH)-containing fibers contact gonadotropin-releasing hormone (GnRH) neuron soma in the preoptic area and terminals in the median eminence (Jennes et al., 1983; Leranth et al., 1988; Kuljis and Advis, 1989). Both the tubero-infundibular dopaminergic pathway (Leranth et al., 1988; Mitchell et al., 2003) and the anteroventral periventricular nucleus (AVPV; Horvath et al., 1993) contribute to the TH innervation of GnRH-secreting neurons. Notably, the kisspeptin neuron subpopulation in the arcuate nucleus (ARC) acts on the tubero-infundibular dopaminergic pathway (Ribeiro et al., 2015) and the sexually dimorphic kisspeptin neuron subpopulation in the AVPV coexpresses TH (Simerly et al., 1985; Clarkson and Herbison, 2011), indicating that dopamine may influence GnRH neuronal activity both directly and indirectly. Throughout the brain, dopamine modulates neuronal excitability (Beaulieu et al., 2015). However, dopamine hyperpolarizes GnRH neurons, via the activation of potassium channels (Liu and Herbison, 2013).

Dopamine receptors, based on their structural, pharmacological, and signaling properties, are divided into two families of G-protein-coupled receptors: D1-like (D1 and D5 subtypes) and D2-like (D2, D3, and D4 subtypes; Beaulieu et al., 2015). D1-like receptors couple to Gα_s_ protein, through which they activate adenylyl cyclase, inhibit potassium channels and increase neuronal excitability while D2-like receptors couple to Gα_i/o_ protein and have the opposite effect (Beaulieu et al., 2015). Consistent with this, in fish, dopamine inhibits GnRH neurons via D2-like receptors (Bryant et al., 2016). However, in mouse, both D1-like and D2-like receptors have been reported to contribute to the inhibition (Liu and Herbison, 2013). The current study aimed to identify dopamine receptor subtype(s) regulating GnRH neurons and to elucidate how Gα_s_ protein-coupled D1-like receptors could possibly inhibit GnRH neurons. Our microarray dataset from GnRH neurons in explants revealed a high level of *Drd4* transcripts coding for dopamine D4 receptor (D4R) compared with other dopamine receptors, suggesting a potential preferential role in GnRH neurons. The present results demonstrate that, while each GnRH neuron did not express all five dopamine receptors, all dopamine receptors were present within the GnRH neuron population. In primary mouse GnRH neurons maintained in explants, devoid of kisspeptin neurons and TH containing neurons, calcium imaging revealed that activation of D1-like receptors only increased GnRH neuronal activity, via G_αs_ protein coupling. In contrast, activation of D2-like receptors, including D4R, decreased GnRH neuronal activity, via G_αi/o_ protein coupling. Consistent with these results, patch clamp revealed D4R specific activation decreased GnRH neuron firing in brain slices. These data demonstrate that dopamine inhibits GnRH neuronal activity via activation of D2-like receptors and identifies D4R as a new contributor to this inhibition.

## Materials and Methods

### Nasal explants

All procedures were conducted in accordance with the Society for Neuroscience’s Policies on the Use of Animals and Humans in Research. Unsexed embryos from timed mated NIH Swiss mice were used to generate nasal explants, as previously described (Klenke and Taylor-Burds, 2012). One embryo gives one explant. Explants were maintained at 37°C in serum-free medium (SFM) in a 5% CO_2_ humidified incubator. Fresh media containing fluorodeoxyuridine (2.3 μm; Sigma-Aldrich) was applied on culture day 3 to inhibit proliferation of dividing olfactory neurons and nonneuronal explant tissue. On culture day 6, and every other day afterward, the medium was changed with fresh SFM. After days 2–3 *in vitro*, GnRH neurons migrate out of the explant and can be recorded in the periphery.

### Single GnRH cell isolation and microarray data

This dataset was previously generated (Messina et al., 2011). Briefly, the cytoplasmic content of single GnRH neurons in 10-d *in vitro* explants was extracted and poly(A) amplified cDNA libraries were generated (Kramer, 2002).

cDNA libraires from nine individual GnRH neurons 10 div (see below) were randomly grouped into three samples. This material was amplified, processed for microarray data generation and analysis. All cDNA were labeled and hybridized by the DIRP NIH microarray core facility with GeneChip Mouse Genome 430 2.0 arrays (Affymetrix). Custom R scripts, including covariance-based PCA, correlation heat maps, LOWESS analysis, and clustering checked the dataset for noise and outliers. Normalization, i.e., the average expression of genes, was performed identically throughout the dataset and log-transformed. Gene expression values were calculated using robust multiarray average (RMA) procedure and compared with repeated-measurement one-way ANOVA, using data from one probe set (Drd1_629) as a reference.

### PCR on single cells and explants

Poly(A) amplified cDNA libraries were previously created from whole explants and individual GnRH cells in explants (Kramer, 2002). The quality of cDNA from each cell was verified by PCR for GnRH, L19, and β-tubulin (Giacobini et al., 2004). Primers were designed in the 3′-untranslated region of genes Drd1, Drd2, Drd3, Drd4, and Drd5 (Table 1). All primers were screened with BLAST to ensure specificity. For each reaction, 1× PCR buffer, 2 mm MgCl_2_, 250 μm of each deoxynucleotide; Life Technologies), 250 nm forward primer, 250 nm reverse primer, and 2.5 U Ampli-Taq Gold (Life Technologies) were combined with 1-μl template cDNA. PCR for Drd1 and Drd2 was performed as follows: initial 10-min denaturation (94°C), 40 cycles of 30-s denaturation (94°C), 30-s annealing (55°C), and 2-min extension (72°C), followed by a 10 min after elongation (72°C). PCR for Drd3, Drd4, and Drd5 used the same steps, but included a “touch-up” annealing temperature with 0.1°C increment per cycle for the first 20 cycles (55–58°C, shown in Table 1), followed by 30 cycles at the highest annealing temperature (57–60°C). PCR for Drd3 and Drd5 used a “nested” technique in which PCR product from an initial reaction was used as template in a subsequent reaction with the same protocol and different primers. Table 1 shows the annealing temperature of the 40 cycles (Drd1, Drd2) and the annealing temperature range of the 20 cycles and the annealing temperature of the subsequent 30 cycles (Drd3, Drd4, Drd5) and the product size for each primer combinations. Amplified products were run on a 1.5% agarose gel. Specific bands of the predicted size were observed in control brain, whereas no bands were seen in water.

**Table 1:** Primer sequences

Gene (NCBI/GenBank reference sequence)	Primers sequences (5′−3′)	Annealing temperature	Product size
D1 dopamine receptor (Drd1) (NM_001291801.1, NM_010076.3)	F: GTACCATCAAGTCCCCTCGG	55°C (40×)	120 bp
	R: CAGCCCTTCCTTCAGTTCTATC

D2 dopamine receptor (Drd2) (XM_006509996.3)	F: GCTGAAGTTGGAGGTGGTAA	55°C (40×)	145 bp
	R: CCAGACCCAATGGTATCAGCA

D3 dopamine receptor (Drd3) (NM_007877.2, XM_006521777.2) *Nested: F2/*R*2	F1: GGCCTTCATTGTCTGTTGGC	57.5°C → 59.5°C (20×, +0.1°C/cycle) + 59.5°C (30×)	228 bp
	R1: AAGTGGGTAAAGGGAATGTCTC
	F2: CCTTCTTCTTGACTCACGTTCT	58°C → 6°C (20×, +0.1 C/cycle) + 6°C (30×)	158 bp
	*R*2: CTTGAGGAAGGCTTTGCGGA

D4 dopamine receptor (Drd4) (XM_006536156.3, XM_017321972.1)	F: TCTCTGGAAGCTTGGGAAACT	55°C → 57°C (20×, +0.1°C/cycle) + 57°C (30×)	284 bp
	R: GGCAGGAAACAAGACCAAA

D5 dopamine receptor (Drd5) (NM_013503.3) *Nested: F1/*R*2	F1: GAGTACGGTGAAGTGTCCTTTAT	55°C → 57°C (20×, +0.1°C/cycle) + 57°C (30×)	245 bp
	R1: GAGTACGGTGAAGTTCCTTTAT
	*R*2: CGGCTGTTCAGAAGACTCATAA	55°C → 57°C (20×, +0.1°C/cycle) + 57°C (30×)	202 bp

### Calcium imaging

**Figure 1.**
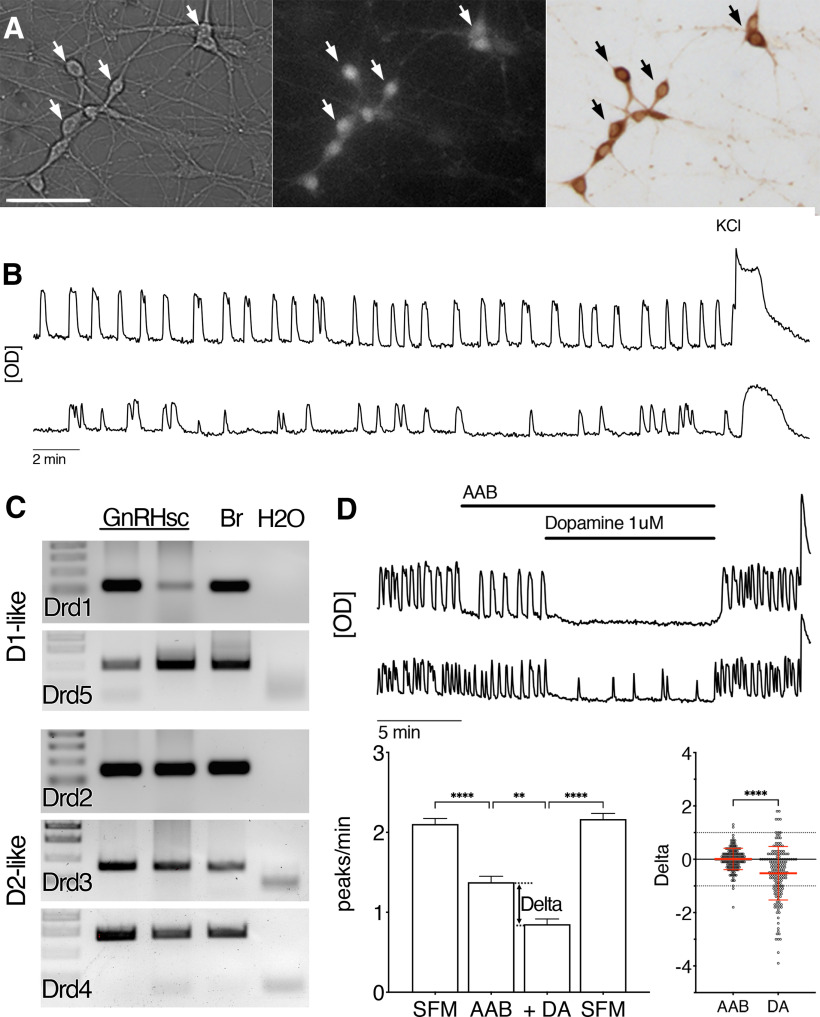
GnRH neurons respond to dopamine. ***A***, GnRH neurons obtained from an E11.5 mouse and maintained in culture for 7 d recorded for calcium imaging. GnRH cells (arrows) were identified under brightfield by their bipolar morphology (left panel), recorded after loading with Calcium Green 1-AM (middle panel), and then stained for GnRH after recording (right panel). Scale bar: 50 μm. ***B***, Representative calcium imaging recording of two single GnRH neurons showing spontaneous intracellular calcium oscillations (peaks) in SFM. Recordings were terminated with 40 mm KCl to ensure viability of cells. *y*-axis, arbitrary OD units; *x*-axis, 2 min. ***C***, Analysis of single GnRH neuron cDNA libraries show all five dopamine receptors, dopamine D1-like receptors (D1 and D5) and dopamine D2-like dopamine receptors (D2, D3, and D4), within the GnRH neuronal population. GnRHsc, GnRH single cells; Br, brain; H20, water. ***D***, Representative calcium imaging recordings of two GnRH neurons in AAB (20 μm BIC, 10 μm CNQX, and 10 μm AP5) plus dopamine (1 μm). The rate of spontaneous calcium oscillations in GnRH neurons was reduced after blocking the GABAergic and glutamatergic excitatory inputs, then further inhibited with dopamine, indicating a direct effect of dopamine on GnRH neuronal activity. Bar graph (mean ± SEM) indicates the quantification in peaks/min from all cells (*n* = 162, *N* = 6). Nonparametric Friedman tests, followed by Dunn’s multiple comparisons test with significance shown using GraphPad style: *0.01 < *p* < 0.05, **0.001 < *p* < 0.01, ***0.0001 < *p* < 0.001, ****p < 0.0001. Beeswarm plot indicates the spread of individual cell changes in the frequency of calcium oscillations in response to dopamine (DA) compared with the spread of individual cell spontaneous changes when maintained in AAB (mean ± SD). The δ values are calculated at the drug switch highlighted on the bar graph. Nonparametric Kruskall–Wallis, followed by Dunn’s multiple comparisons test, was used with DA as the reference.

Calcium imaging recordings were performed on explants between 6 and 11 d *in vitro* (d) as previously described (Klenke and Taylor-Burds, 2012; Fig. 1*A*,*B*). Briefly, cells were loaded with 13.5 μm Calcium Green-1 for 20 min at 37°C in a 5% CO_2_ humidified incubator, then washed for 20 min in fresh SFM. After loading, explants were mounted into a perfusion chamber (Warner Instruments) and continuously perfused with SFM at a rate of 300 μl/min using a peristaltic pump (Instech). All experiments had a control period (SFM; 5 min), amino acid blocking period (AAB; 5 min), one or two drug treatment periods (5 or 10 min each), and a final washout period (SFM; 5 min). Time-lapse images piloted by imaging software (BioVision) were taken of cells every 2 s for up to 30 min. Images were obtained through a 20× fluorescence objective using an inverted Nikon microscope and a charge-coupled device camera (QImaging) connected to a computer. Excitation wavelengths of 465–495 nm were provided through a medium-width excitation bandpass filter, and emission was monitored through a 40-nm bandpass centered on 535 nm. All recordings were completed with a 40 mm KCl stimulation to ensure viability of cells. Changes in levels of gray over time [optical density (OD), arbitrary unit] were quantified in single GnRH neurons a posteriori with iVision and calcium oscillations, reflecting neuronal activity (Constantin and Wray, 2008), detected with MATLAB (MathWorks). The frequency of oscillations was expressed in peaks per minute. The phenotype of cells included in the results was confirmed by immunocytochemistry using anti-GnRH primary antibody previously described (Wray et al., 1989).

### Electrophysiology

Loose patch clamp was performed on GnRH neurons from acute brain slices. Since sex hormone (estradiol, progesterone) fluctuations during the estrous cycle in females are known to influence GnRH neuron excitability (Liu and Herbison, 2011; Farkas et al., 2013; Silveira et al., 2017; Adams et al., 2018), male mice were used for slice recording (Lee et al., 2012; Herde et al., 2013; Constantin and Wray, 2018; Constantin et al., 2021a, b).

GnRH-GFP mice (MGI:6158458; Spergel et al., 1999) were killed at ∼10:30 A.M. by cervical dislocation then decapitated. The brain was glued to a vibratome plate and submerged into ice-cold low [Ca^2+^]/high [Mg^2+^] (0.5/6 mm, respectively) artificial CSF (aCSF), bubbled with 95% O_2_/5% CO_2_. After vibratome sectioning (Leica VT1000S), coronal 200-μm slices were incubated at 30°C in normal aCSF containing the following: 118 mm NaCl, 3 mm KCl, 2.5 mm CaCl2, 1.2 mm MgCl_2_, 10 mm HEPES, 25 mm NaHCO_3_, and 11 mm D-glucose (pH 7.3), bubbled with 95% O_2_/5% CO_2_.

Slices were transferred into a recording chamber mounted on an upright microscope (Nikon Eclipse FN1) and continuously perfused with oxygenated normal aCSF maintained at 28–30°C, at a rate of ∼2 ml/min (Constantin et al., 2012). GnRH neurons were identified in slices under fluorescence (20-nm narrow bandpass EGFP filter centered at 480 nm) using a 40× water immersion objective (Nikon 40×/0.80 W, WD 2.0). GnRH neurons were patched under differential interference contrast through a charge-coupled device camera (QImaging Retiga EXi Blue) piloted by the open-source software Micro-Manager version 1.4. The pipettes (3–5 MΩ) were backfilled with aCSF. Electrophysiological recordings were acquired with a Multiclamp 700B amplifier (Molecular Devices) using a lowpass filter at 10 kHz and digitized by a Digidata (1550) analog-to-digital converter at 10 kHz (Molecular Devices).

### Analysis

For calcium imaging, nonparametric Friedman tests, followed by Dunn’s multiple comparisons test, were used to compare peaks/min of cells between treatment periods of an experiment (Fig. 1*D*, bar plot). The magnitude of the effect evoked by an agonist was calculated for each cell as the difference (δ) between the frequency of calcium oscillations during the period before the agonist and the frequency of calcium oscillations during the period with agonist. Nonparametric Mann–Whitney rank tests were used to compare δ between two paradigms only and nonparametric Kruskall–Wallis tests, followed by Dunn’s multiple comparisons test, were used to compare δ of the effects between more than two paradigms. A response, inhibition or excitation, to a pharmacological challenge was defined as δ < −1 or δ > 1 peak/min, respectively.

For electrophysiology, action potentials (APs) were detected with Clampfit 10 on continuous recordings. The average firing frequency was calculated over the last 3 min of two consecutive recording periods. The firing rate during the second period (dopamine application) was normalized to the firing rate during the first period and expressed as a percentage. A response to dopamine was defined as a change in firing >20%. Individual cell changes in firing between two consecutive treatments were analyzed with paired *t* test. Changes in firing rate between two paradigms were analyzed with unpaired *t* test. Significance was determined by *p *<* *0.05, and data are presented as mean ± SEM (*n* and *N*, representing the number of cells and animals used, respectively).

### Drugs

(-)-Bicuculline methochloride (BIC; GABA_A_ receptor antagonist), D-(-)−2-amino-5-phosphonopentanoic acid (AP5; NMDA glutamatergic receptor antagonist), CNQX disodium salt (AMPA/kainate receptor antagonist), A 68930 hydrochloride (D1/5R agonist), SCH-23390 hydrochloride (D1/5R antagonist), (S)-(-)-Sulpiride (D2/D3R antagonist), A 412997 dihydrochloride (D4R agonist), L-745870 trihydrochloride, and PD 168568 dihydrochloride (D4R antagonist) and (-)-quinpirole hydrochloride (D2-like receptor agonist) were obtained from Tocris.

Cholera toxin (CTX; Gα_s_ protein uncoupling agent) and pertussis toxin (PTX; Gα_i/o_ protein uncoupling agent) were obtained from Sigma-Aldrich. Dopamine hydrochloride was purchased from Sigma-Aldrich. (-)-Quinpirole hydrochloride (Sigma-Aldrich) and spiperone (D2-like receptor antagonist; Sigma-Aldrich) were generously provided by David Sibley (National Institutes of Health, National Institute of Neurological Disorders and Stroke). All stock solutions were aliquoted and stored at −20°C in either DMSO or distilled water, and solutions were prepared immediately before each experiment by diluting 1:1000 stock into SFM to minimize oxidation. DMSO (up to 1:500) is known to have no effect on the frequency of calcium oscillations (Constantin et al., 2009). All drugs were applied by perfusion to the explants during imaging except CTX and PTX which were applied for >4 h before recording.

### Immunocytochemistry

After calcium imaging, explants (6–11 d) were fixed for 30 min with 0.1 m PBS pH 7.4 containing 4% formaldehyde at room temperature. After a few washes in PBS, explants were incubated in a blocking solution (10% normal horse serum + 0.3% Triton X-100 + 0.1% NaAzide) for 1 h and washed several times in PBS. The explants were incubated at 4°C overnight in the primary antibody (in PBS with 1% BSA + 0.1% NaAzide; SW-1, Wray et al., 1989; Table 2). The next day, explants were washed in PBS, incubated for 1 h with biotinylated secondary donkey anti-rabbit antibody (1:500 in PBS/0.3% Triton X-100; Vector Laboratories, Inc), washed in PBS, and processed for avidin-biotin horseradish peroxidase/3,3′-diaminobenzidine (Fig. 1*A*).

**Table 2:** Antibody table

Peptide/protein target	Name of antibody	Manufacturer, catalog number, and/or name of individual providing the antibody	Species raised in; monoclonal or polyclonal	Dilution used
rbGnRH	SW-1	S. Wray	Rabbit; polyclonal	1:15,000 (*in vivo*) 1:5000 (*in vitro*)
mGnRH	F1D3C5+SMI-41	A. Karande + Abcam	Mouse; monoclonal	1:4000 +1:6000
GFP	Anti-GFP	Abcam, ab92456	Chicken; polyclonal	1:2000
Dopamine receptor D4	Anti-D4	Abcam, ab135978	Rabbit; polyclonal	1:24,000
	DRD4 clone 2B9	Millipore, MABN125	Mouse; monoclonal	10 μg/ml
	D4 HL7420 AP	A. Buonanno	Rabbit; polyclonal	2 μg/ml
Dopamine receptor D1	clone 1–1-F11	Sigma, D187	Rat; monoclonal	1:100

### Immunofluorescence

Primary antibodies are listed in the antibody table (Table 2).

Explants were fixed for 1 h in 4% formaldehyde. After washing in PBS, explants were incubated in blocking solution (1 h; 10% normal horse serum + 0.3% Triton X-100), washed in PBS, and then incubated in primary antibody (anti-D4 or anti-D1; 2 nights at 4°C). The next day, explants were washed in PBS, incubated in either Alexa Fluor 555-conjugated secondary anti-rabbit antibody for anti-D4 antibody (1 h; 1:1000; Life Technologies) or biotinylated secondary anti-mouse or anti-rabbit antibody, for anti-D4 clone 2B9 and HL7420 AP antibody, respectively, or biotinylated secondary anti-rat antibody for anti-D1 antibody (1 h; 1:500 + 0.3% Triton X-100; Vector Laboratories, Inc). Explants treated with a biotinylated secondary antibody were washed then incubated with Alexa Fluor 555-conjugated avidin (1 h; 1:1000; Life Technologies). After few washes in PBS, explants were briefly fixed, washed, and incubated in a second primary antibody raised in different species (anti-GnRH; one to two nights at 4°C). The next day explants were washed, treated with Alexa Fluor 488-conjugated anti-mouse or anti-rabbit antibody (1 h; 1:1000; Invitrogen) for anti-GnRH F1D3C5+SMI-41 or SW-1 antibody, respectively, washed in PBS, and coverslipped with an antifade mounting solution (Electron Microscopy Sciences). No anti-D4R primary antibody controls were run for each antibody to determine non-specific background staining on GnRH-labeled neurons.

## Results

### All dopamine receptors are detected in prenatal GnRH neurons

The relevance of prenatal GnRH neurons is often questioned when assessing the physiology of adult GnRH neurons. Therefore, 48 genes previously detected in adult GnRH neurons (Todman et al., 2005; Burger et al., 2018) were compared with the microarray dataset obtained from GnRH cells in explants (Table 3). We also included transcripts for 6 genes previously detected by single-cell PCR of GnRH neurons in explants (Sharifi et al., 2002; Klenke et al., 2010; Constantin et al., 2009, 2016; Constantin and Wray, 2018).

**Table 3:** Affymetrix RMA table

Affymetrix probe set ID	Gene name	Full gene name	RMA value 10 d *in vitro*		*P* value	Mouse	
			Mean	SEM		Adult brain slice	Prenatal explant
Dopamine receptors
1418950_at	Drd2	Dopamine receptor 2	3.71	0.15	0.0220	PMID: 15837132, PMID: 29522155	
1422278_at	Drd3	Dopamine receptor 3	2.71	0.21	0.0371		
1422829_at	Drd4	Dopamine receptor 4	6.34	0.21	0.0067		
1422830_s_at	Drd4	Dopamine receptor 4	5.42	0.07	0.0019		
1455629_at	Drd1a	Dopamine receptor D1A	4.30	0.09	Ref	PMID: 29522155	
1456051_at	Drd1a	Dopamine receptor D1A	4.31	0.20	>0.9999	PMID: 29522155	
Neuropeptide/steroid/opioid receptors
1450493_at	Kiss1r	Kiss1 receptor	7.39	0.22		PMID: 29522155	PMID: 18948403
1422099_a_at	Oprl1	Opioid receptor-like 1	4.21	0.27			PMID: 30627649
1426103_a_at	Esr2	Estrogen receptor 2 (β)	5.91	0.09		PMID: 29522155	PMID: 12072381
1428250_at	Gper	G-protein-coupled estrogen receptor 1	4.88	0.15		PMID: 29522155	PMID: 26934298
1422256_at	Sstr2	Somatostatin receptor 2	4.23	0.12		PMID: 15837132	
1441603_at	Sstr3	Somatostatin receptor 3	5.03	0.26		PMID: 15837132	
1422281_at	Sstr4	Somatostatin receptor 4	4.19	0.21		PMID: 15837132	
1449572_at	Trhr	Thyrotropin-releasing hormone receptor	3.34	0.10		PMID: 15837132	
1418810_at	Crhr1	Corticotropin-releasing hormone receptor 1	4.96	0.06		PMID: 15837132	
1422204_at	Avpr1b	Arginine vasopressin receptor 1B	5.05	0.28		PMID: 15837132	
1427704_a_at	Galr1	Galanin receptor 1	3.67	0.18		PMID: 15837132, PMID: 29522155	PMID: 27359210
1422942_at	Galr2	Galanin receptor 2	3.39	0.03		PMID: 15837132	
1426054_at	Npy1r	Neuropeptide Y receptor Y1	2.93	0.08		PMID: 29522155	PMID: 20351316
1417489_at	Npy2r	Neuropeptide Y receptor Y2	2.16	0.08		PMID: 15837132, PMID: 29522155	
1422342_at	Nmbr	Neuromedin B receptor	3.78	0.11		PMID: 15837132	
1450260_at	Grpr	Gastrin releasing peptide receptor	3.08	0.17		PMID: 15837132	
1422265_at	Brs3	Bombesin-like receptor 3	3.72	0.13		PMID: 15837132	
1421667_at	Nmur1	Neuromedin U receptor 1	5.66	0.12		PMID: 15837132	
1422121_at	Oprd1	Opioid receptor, δ 1	5.15	0.28		PMID: 15837132	
1417151_a_at	Ntsr2	Neurotensin receptor 2	4.87	0.10		PMID: 15837132	
1450278_at	Tacr3	Tachykinin receptor 3	4.27	0.11		PMID: 15837132	
1422263_at	Bdkrb2	Bradykinin receptor, β 2	5.56	0.09		PMID: 15837132	
1449160_at	Npr1	Natriuretic peptide receptor 1	4.28	0.17		PMID: 15837132	
1427191_at	Npr2	Natriuretic peptide receptor 2	5.01	0.24		PMID: 15837132	
Glutamate (Glu) receptors
1457003_at	Grin2b	Glu receptor, ionotropic, NMDA2B, epsilon 1	5.42	0.16		PMID: 15837132	
1421393_at	Grin2d	Glu receptor, ionotropic, NMDA2D, epsilon 4	6.99	0.18		PMID: 15837132	
1458285_at	Gria1	Glu receptor, ionotropic, AMPA1, α 1	4.34	0.10		PMID: 15837132	
1420563_at	Gria3	Glu receptor, ionotropic, AMPA3, α 3	5.35	0.07		PMID: 15837132	
1421569_at	Grid1	Glu receptor, ionotropic, δ 1	4.67	0.01		PMID: 15837132	
1435487_at	Grid2	Glu receptor, ionotropic, δ 2	2.65	0.22		PMID: 15837132	
1456119_at	Grm5	Glu receptor, metabotropic 5	3.13	0.31		PMID: 15837132	
1421530_a_at	Grm8	Glu receptor, metabotropic 8	3.23	0.24		PMID: 15837132	
GABA receptors
1443865_at	Gabra2	GABA receptor A, α 2	4.24	0.26		PMID: 15837132	
1419719_at	Gabrb1	GABA receptor A, β 1	4.63	0.24		PMID: 15837132	
1450319_at	Gabrb2	GABA receptor A, β 2	3.28	0.20		PMID: 15837132	
1450300_at	Gabrr1	GABA receptor C, ρ 1	3.07	0.07		PMID: 15837132	
Other amine receptors
1450003_at	Adra2b	Adrenergic receptor, α 2b	5.13	0.10		PMID: 15837132	
1423420_at	Adrb1	Adrenergic receptor, β 1	2.90	0.13		PMID: 15837132	
1438710_at	Htr1a	5-Hydroxytryptamine (serotonin) receptor 1A	4.63	0.29		PMID: 15837132	
1418268_at	Htr3a	5-Hydroxytryptamine (serotonin) receptor 3A	3.99	0.22		PMID: 15837132	
1427654_a_at	Htr4	5-Hydroxytryptamine (serotonin) receptor 4	4.68	0.15		PMID: 15837132	
1419210_at	Hrh1	Histamine receptor H1	4.12	0.25		PMID: 15837132	
1423639_at	Hrh2	Histamine receptor H2	6.07	0.10		PMID: 15837132	
Cholinergic receptors
1439611_at	Chrm1	Cholinergic receptor, muscarinic 1, CNS	4.69	0.11		PMID: 15837132	
1420682_at	Chrnb1	Cholinergic receptor, nicotinic, β 1	2.43	0.10		PMID: 15837132	
1420744_at	Chrnb2	Cholinergic receptor, nicotinic, β 2	5.16	0.04		PMID: 15837132	
1449532_at	Chrng	Cholinergic receptor, nicotinic, γ	6.54	0.15		PMID: 15837132	

The RMA values for transcripts for Drd1, Drd2, Drd3, and Drd4 dopamine receptors ranged between 2.71 and 6.34. Compared with the gene expression values from the probe set Drd1_629, gene expression values for both probe sets for Drd4 were higher, while gene expression values for Drd2 and Drd3 were lower. Analysis of cDNAs generated from single GnRH neurons (*n* = 5–7) demonstrated that transcripts for each dopamine receptor are present in a subset of GnRH neurons (Fig. 1*C*).

### Exogenous dopamine directly reduces GnRH neuron activity

Exogenous dopamine (1 μm) was applied to explants and changes in frequency of intracellular calcium in GnRH neurons were determined using calcium imaging. Since GABAergic and glutamatergic inputs to GnRH neurons are robust in explants (Constantin et al., 2010), excitatory inputs were blocked by treatment with AABs (20 μm BIC, 10 μm CNQX, 10 μm AP5). After a control period (SFM 5 min, followed by AAB 5 min), dopamine was added (Fig. 1*D*, top). Dopamine decreased the mean frequency of calcium oscillations in GnRH neurons, indicating mainly inhibition (Fig. 1*D*, bottom left). Using ±1 peaks/min as cutoff values in changes in the frequency of calcium oscillations, ∼25% of the GnRH cells were inhibited by dopamine (δ < −1) while ∼5% were excited (δ > 1; Fig. 1*D*, bottom right).

### The dopamine D2-like receptor, D4R, is required for the dopamine inhibition

**Figure 2.**
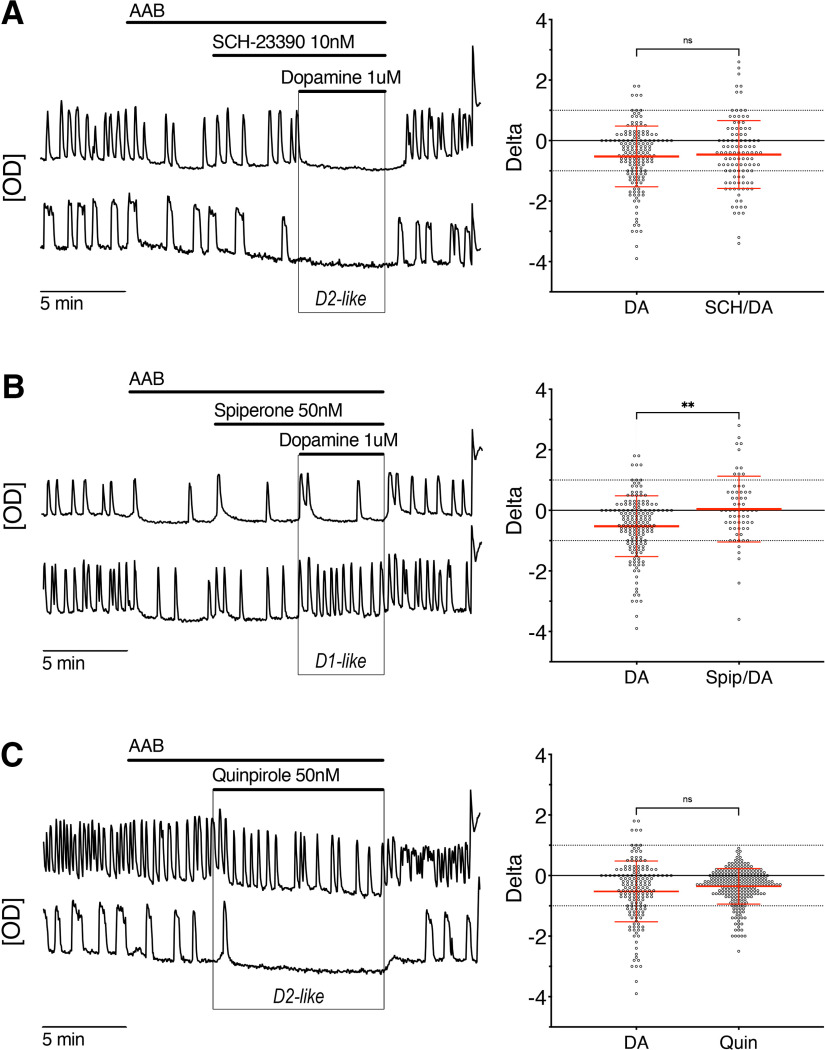
The inhibitory effect of dopamine is mediated through the dopamine D2-like receptor subfamily. ***A–C***, Representative calcium imaging recordings (left, OD units) and calculated δ values for all GnRH cells in that experimental group in beeswarm plot (mean ± SD; right). ***A***, Blocking D1/5R (SCH-23390 10 nm) did not prevent the inhibitory effect of dopamine (1 μm; *n* = 111, *N* = 3). ***B***, In contrast, a D2-like receptor antagonist (spiperone 50 nm) blocked the inhibitory effect of dopamine. Notably, while the numbers of cells showing inhibition decreased (∼25% down to ∼8%) by spiperone + dopamine (left, top trace), the numbers of cells showing excitation increased (∼5% up to ∼16%, right, bottom trace and beeswarm plot; *n* = 64, *N* = 3). ***C***, The inhibition could be evoked with a dopamine D2-like receptor agonist (quinpirole 50 nm; *n* = 238, *N* = 6). Combined, these data suggest that the inhibitory effect of dopamine is mediated via the dopamine D2-like receptor subfamily. Beeswarm plot indicates the spread of individual cell changes in the frequency of calcium oscillations in response to dopamine (DA; from Fig. 1*D*) compared with the spread of individual cell changes when challenged with antagonist/DA or agonist. Nonparametric Kruskall–Wallis, followed by Dunn’s multiple comparisons test, was used with DA as the reference.

Canonically, the D1-like subfamily of dopamine receptors couple to the stimulatory Gα_s_ protein subunit while the D2-like subfamily couple to the inhibitory Gα_i/o_ protein subunit. Thus, the decrease in GnRH neuronal activity after exposure to dopamine suggested the activation of D2-like receptors. Consistent with this observation, dopamine-mediated inhibition persisted in presence of the D1-like receptor antagonist SCH-23390 (10 nm; Fig. 2*A*, traces) and the magnitudes of the dopamine inhibition, with or without SCH-23390, were the same (Fig. 2*A*, beeswarm plot) suggesting a minimal role of D1-like receptors, if any. Conversely, dopamine-mediated inhibition was abolished in presence of D2-like receptor antagonist spiperone (50 nm; Fig. 2*B*, beeswarm plot). Notably, while the numbers of cells showing inhibition decreased (∼25% down to ∼8%) by spiperone + dopamine (Fig. 2*B*, top trace), the numbers of cells showing excitation increased (∼5% up to ∼16%; Fig. 2*B*, bottom trace). Consistent with the SCH-23390/dopamine experiment, the D2-like receptor agonist quinpirole (50 nm) decreased the frequency of calcium oscillations in GnRH neurons (Fig. 2*C*, traces) and the magnitudes of the quinpirole and dopamine inhibition were the same (Fig. 2*C*, beeswarm plot).

**Figure 3.**
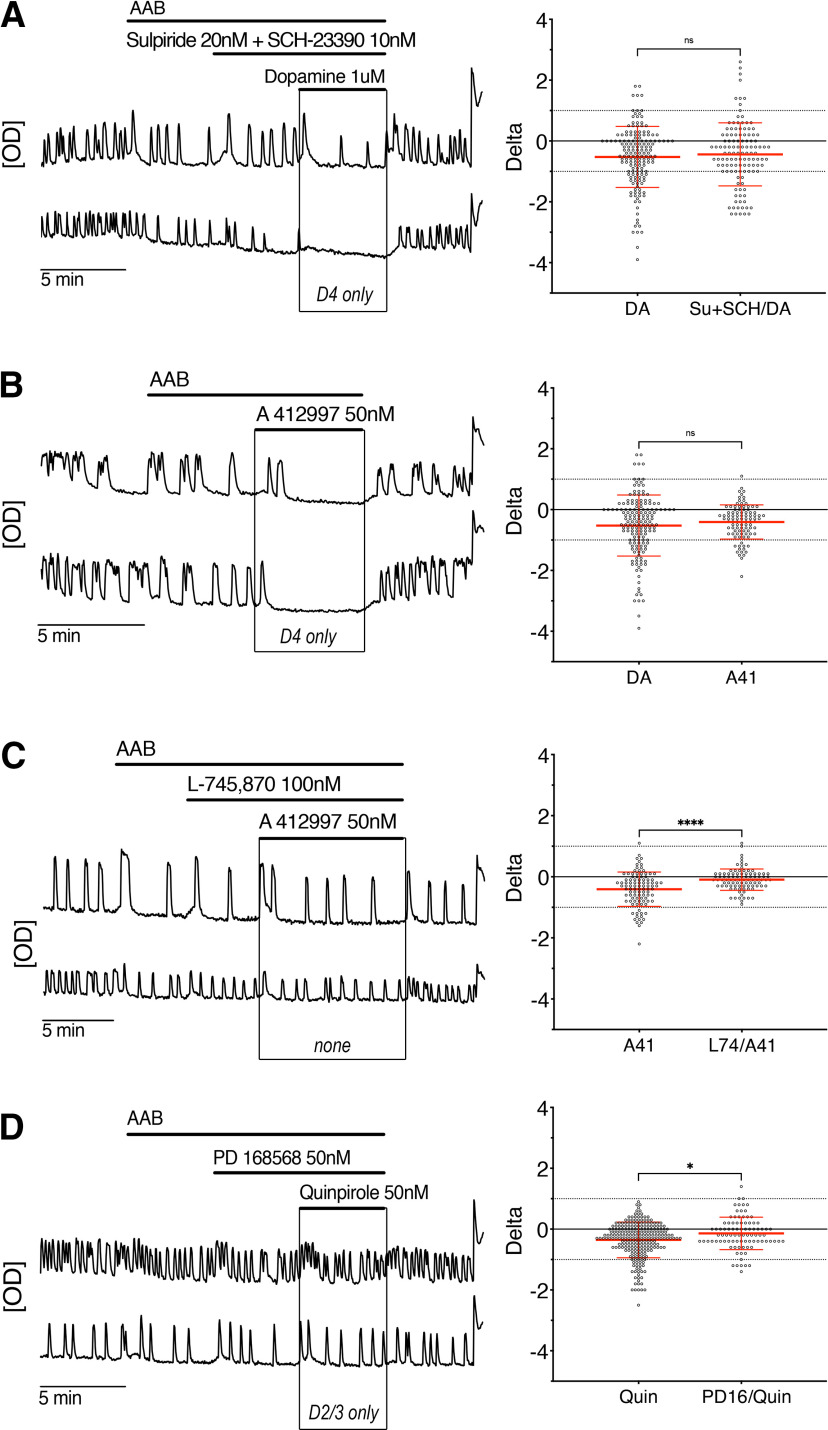
Dopamine-mediated inhibition of GnRH neurons is dependent on dopamine D4 receptor. ***A–D***, Representative calcium imaging recordings (left, OD units) and calculated δ values for all GnRH cells in that experimental group in beeswarm plot (mean ± SD; right). ***A***, Blocking D2/D3R (sulpiride 20 nm) did not abolish the dopamine-induced inhibition of GnRH neurons (*n* = 120, *N* = 4). ***B***, Activation of D4R using a specific agonist (A 412997 50 nm) inhibited GnRH neuronal activity (*n* = 99, *N* = 3). ***C***, Co-application of a D4R antagonist (L-745870 100 nm) prevented the effect of the D4R agonist, demonstrating the specificity of A412997 (*n* = 93, *N* = 3). ***D***, Application of another D4R antagonist (PD 168568 50 nm) also prevented the inhibitory effect of the D2-like receptor agonist (quinpirole 50 nm) in GnRH neurons, indicating that the inhibitory effect of quinpirole is mostly mediated through D4R (*n* = 89, *N* = 3). Beeswarm plot indicates the spread of individual cell changes in the frequency of calcium oscillations in response to dopamine (DA; from Fig. 1*D*) compared with the spread of individual cell changes when challenged with antagonist/DA or agonist. Nonparametric Kruskall–Wallis, followed by Dunn’s multiple comparisons test, was used with DA as the reference (***A***, ***B***) or nonparametric Mann–Whitney rank test when only using two samples were compared (***C***, ***D***).

Since the three members of the D2-like receptor family were found by PCR, we used pharmacology to determine which receptor(s) transduced the dopamine-mediated inhibition. In presence of the D2/3R antagonist, sulpiride (20 nm) and SCH-23390 (D1/5R antagonist), dopamine still inhibited the activity of GnRH neurons (Fig. 3*A*, traces) with the same magnitude as dopamine alone (Fig. 3*A*, beeswarm plot), indicating a major role for D4R in the inhibition. Accordingly, the D4R agonist A 412997 (50 nm) alone decreased the frequency of calcium oscillations in GnRH neurons (Fig. 3*B*, traces). About ∼14% of the GnRH cells were inhibited by A 412997 while ∼1% were excited. The magnitude of the inhibition with A 412997 was similar to the one with dopamine (Fig. 3*B*, beeswarm plot). To ensure the subtype specificity of A 412997, the D4R antagonists L-745870 (100 nm) was co-applied with A 412997 and the inhibition was prevented (Fig. 3*C*, traces and beeswarm plot). Finally, to test the role of D2/3R in the dopamine-mediated inhibition, quinpirole (D2-like agonist) was applied in presence of another D4R antagonist PD 168568 (50 nm), also effective on the A 412997-mediated inhibition, to assess the contribution of D4R in the D2-like-mediated inhibition. The inhibition was partially prevented by the D4R antagonist (Fig. 3*D*, traces and beeswarm plot). Together, the data indicate that dopaminergic inhibition of GnRH neurons required the activation of D4R.

### The dopamine D1-like receptor activates GnRH neuronal activity

**Figure 4.**
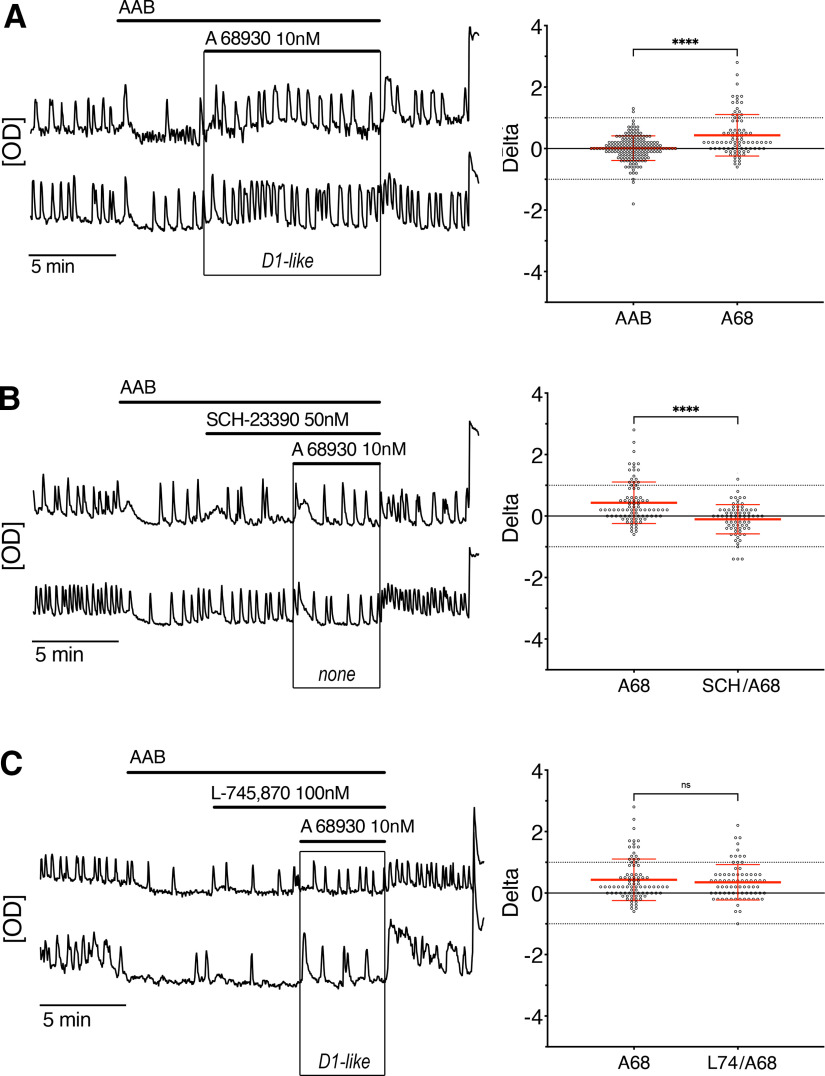
Dopamine D1-like receptor activation increases the activity of GnRH neurons. ***A–C***, Representative calcium imaging recordings (left, OD units) and calculated δ values for all GnRH cells in that experimental group in beeswarm plot (mean ± SD; right). ***A***, Contrary to the inhibitory effect of dopamine, application of a D1-like receptor agonist (A 68930 1 nm) increased the rate of spontaneous calcium oscillations in GnRH neurons (*n* = 86, *N* = 3). ***B***, The stimulatory effect of A 68930 was blocked by the D1-like receptor antagonist (SCH-23390 50 nm), demonstrating the specificity of A 68930 (*n* = 77, *N* = 3). ***C***, Blocking the D4R (L-745870 100 nm) did not prevent the excitatory effect of SCH-23390 (*n* = 77, *N* = 3). Beeswarm plot indicates the spread of individual cell changes in the frequency of calcium oscillations in response to A 68930 compared with the spread of individual cell changes when maintained in AAB or challenged with antagonist/A 68930. Nonparametric Kruskall–Wallis, followed by Dunn’s multiple comparisons test, was used with A 68930 as the reference.

The D1-like receptor agonist A 68930 (10 nm) was applied to test the function of D1/5R. The frequency of calcium oscillations in GnRH neurons increased (Fig. 4*A*, traces). No GnRH cells were inhibited by A 68930 while ∼21% were excited (Fig. 4*A*, beeswarm plot). The co-application of A 68930 and SCH-23390 (D1/5R antagonist) prevented the increase of GnRH neuronal activity (Fig. 4*B*, traces and beeswarm plot) but co-application of A 68930 and L-745870 (D4R antagonist) did not (Fig. 4*C*, traces and beeswarm plot), validating a D1/5R-mediated excitation. These data indicate that dopaminergic inhibition of GnRH neurons did not require D1/5R activation and was entirely mediated through D2-like receptors, specifically D4R.

### D4R triggered Gα_i/o_ protein signaling while D1-like receptors triggered Gα_s_ protein signaling

**Figure 5.**
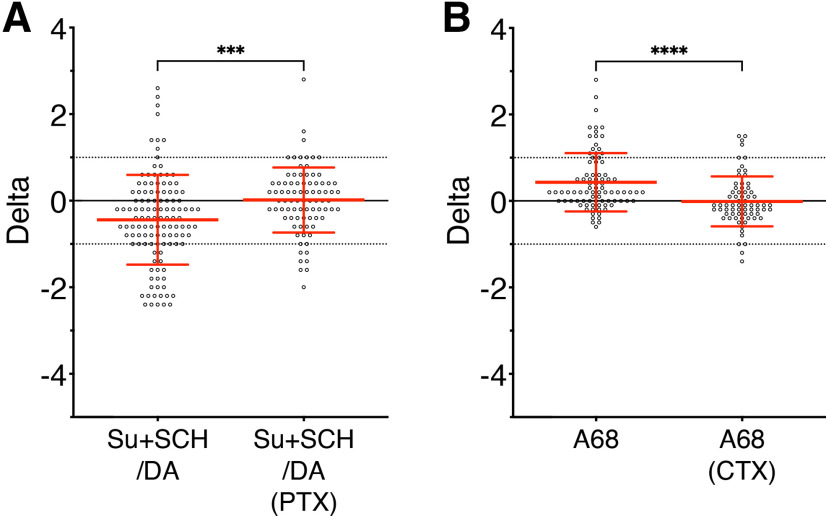
The D1-like and D2-like subfamilies have opposite effects on GnRH neuronal activity via different signaling pathways. ***A***, ***B***, Calculated δ values for all GnRH cells in that experimental group in beeswarm plot (mean ± SD). Control experiments (right), perturbation experiments (left). Control from Figure 3*A* in ***A*** showing D4R activation [dopamine (1 μm) + D1/5R antagonist (SCH-23390 50 nm) + D2/3R antagonist (sulpiride 20 nm)] and control from Figure 4*A* in ***B*** showing D1-like receptor activation [D1/5R agonist (A 68930 1 nm)]. ***A***, D4R activation failed to inhibit GnRH neurons incubated in PTX (>4 h, 250 ng/ml, wrench symbolizes uncoupling Gα_i/o_), demonstrating Gα_i/o_-mediated D4R inhibition (*n* = 84, *N* = 3). ***B***, D1/5R activation failed to excite GnRH neurons incubated in CTX (>4 h, 10 ng/ml, wrench symbolizes uncoupling Gα_s_), demonstrating Gα_s_-mediated D1/5R inhibition (*n* = 70, *N* = 3). Beeswarm plot indicates the spread of individual cell changes in the frequency of calcium oscillations in response to sulpiride+SCH-23390/DA or A 68930 compared with the spread of individual cell changes when pretreated with PTX or CTX, respectively. Nonparametric Mann–Whitney rank test was used to compare two samples (***A***). Nonparametric Kruskall–Wallis, followed by Dunn’s multiple comparisons test, was used with A 68930 as the reference (***B***).

Canonically, the D2-like subfamily of dopamine receptors couples to the inhibitory Gα_i/o_ protein subunit (Beaulieu et al., 2015). Explants were treated with PTX (250 ng/ml) for >4 h to uncouple Gα_i/o_ proteins from their receptors. The D4R was selectively activated using a cocktail of subtype specific antagonists (SCH-23390 for D1/5R and sulpiride for D2/3R) with dopamine. Compared with previous observation, the PTX pretreatment prevented the dopamine inhibition (Fig. 5*A*, beeswarm plot), confirming the Gα_i/o_-mediated inhibition. Since the D1-like subfamily of dopamine receptors couples to the excitatory Gα_s_ protein subunit (Beaulieu et al., 2015), another group of explants were treated with CTX (10 ng/ml) for >4 h to uncouple the Gα_s_ protein from their receptors. Compared with previous observation, CTX pretreatment prevented the excitatory effect of the D1-like receptor agonist A 68930 (Fig. 5*B*, beeswarm plot), supporting Gα_s_-mediated excitation.

### The dopamine D4R is present in GnRH neurons

**Figure 6.**
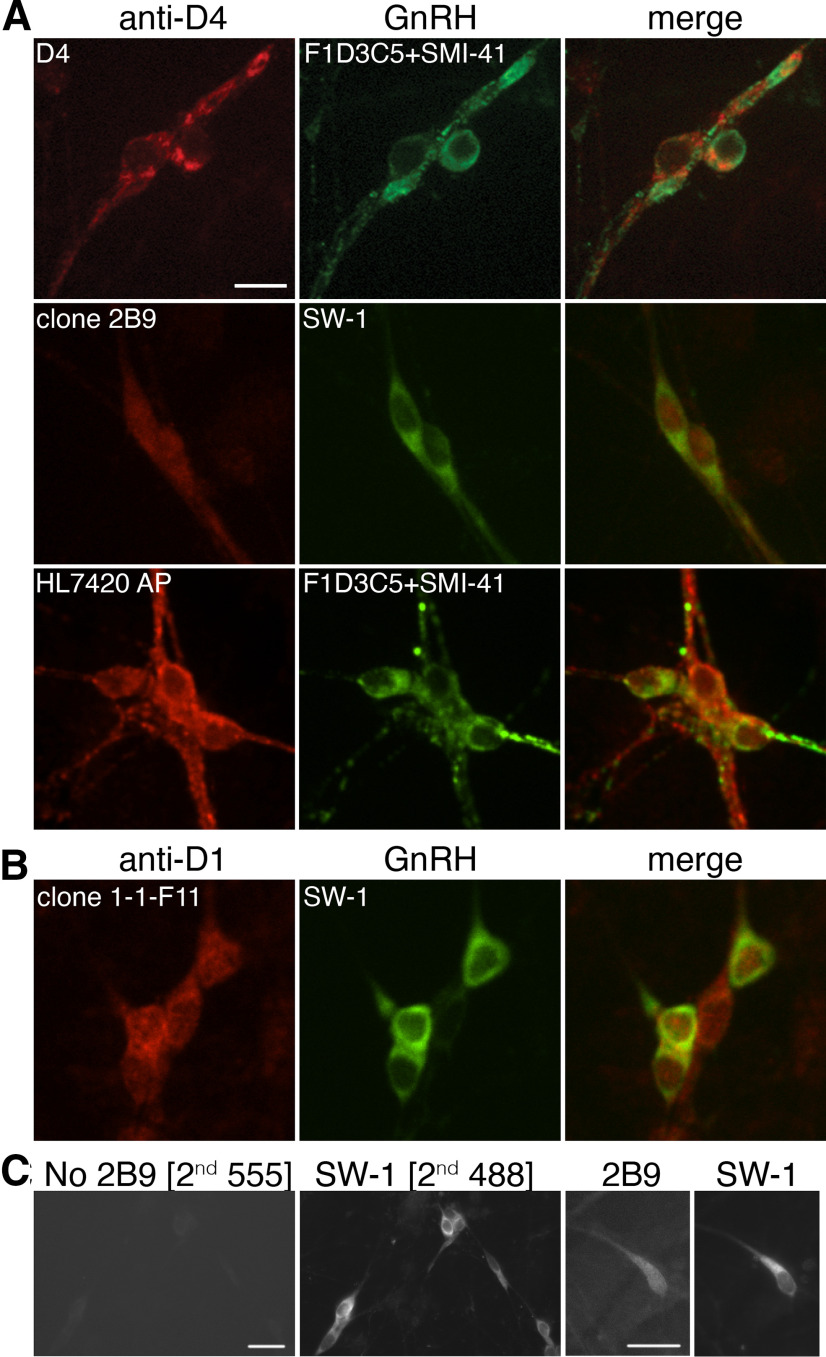
GnRH neurons are immunoreactive for dopamine D4 receptor and dopamine D1 receptor. ***A***, Single confocal plans showing GnRH neurons (green), colabeled with D4R (red; scale bar: 10 μm) in one-week-old explants (*N* = 3), using three different primary antibodies. ***B***, Single confocal plans showing GnRH neurons (green), colabeled with D1R (red; scale bar: 10 μm) in one-week-old explants (*N* = 3). ***C***, Example of control for no primary antibody 2B9 (left) versus primary antibody 2B9 (right) during double label immunofluorescent staining (images from conventional fluorescent microscope, 40×; scale bar: 20 μm).

To support pharmacological data of D4Rs inhibiting GnRH neuronal activity, three different antibodies raised against the D4R were used and confirmed in single plan confocal images, that GnRH neurons in explants express the protein (Fig. 6*A*). The presence of D1R was also confirmed with immunostaining (Fig. 6*B*). No staining was present when the primary antibodies were omitted (Fig. 6*C*, nonconfocal image).

### The dopamine D4R is functional in adult GnRH neurons from acute brain slices

**Figure 7.**
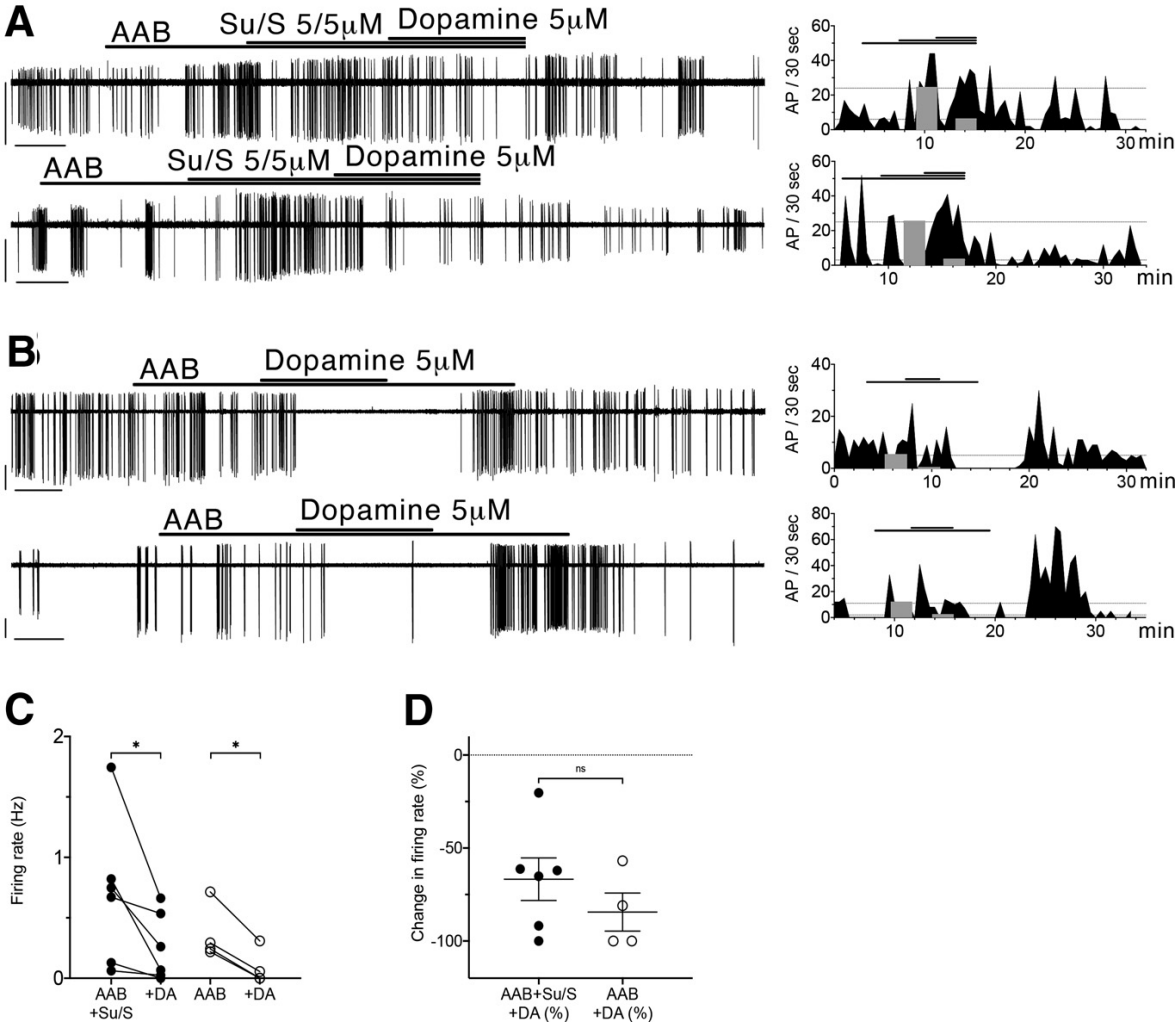
Dopamine D4 receptor activation inhibits GnRH neuron firing. Electrophysiological recording of adult GFP-tagged GnRH neurons and corresponding periodograms showing that dopamine (5 μm) inhibits GnRH neuron firing rate, with or without D1/5R and D2/3R antagonists (***A***, ***B***). The gray bars on the periodograms indicate where the mean firing rates (pre and during dopamine application) were calculated for statistical analysis. ***C***, Summary data showing quantification of firing rate (in Hz) with dopamine (DA) in individual GnRH neurons, with (closed circles) or without (opened circles) D1/5R and D2/3R antagonists. The values represent the average firing rate over the last 3 min of each period. Individual cell changes in firing between two consecutive treatments were analyzed with paired *t* test. ***D***, Summary data showing respective changes in firing rate, with (closed circles) or without (opened circles) D1/5R and D2/3R antagonists. Changes in firing rate between two paradigms were analyzed with unpaired *t* test. Significance is shown using GraphPad style: *0.01 < *p* < 0.05, **0.001 < *p* < 0.01, ***0.0001 < *p* < 0.001, ****p < 0.0001.

D4R activation was next analyzed in GnRH cells in adult brain slices. Similar to that shown in Figure 3*A* for GnRH cells maintained in explants, blocking D1/5R and D2/3R with SCH-23390 (S; 5 μm) and sulpiride (Su; 5 μm), respectively, then applying dopamine (5 μm; Fig. 7*A*) resulted in inhibition of neuronal activity in GnRH cells in brain slices. The effect of dopamine, with D1/5 and D2/3R antagonism, was compared with the effect of dopamine alone (Fig. 7*B*). Six out of 8 cells (six animals) showed inhibition with dopamine after D1/5R and D2/3R antagonists. The firing rate went from 0.70 ± 0.25 to 0.26 ± 0.12 Hz. Four out of five cells (four animals) showed inhibition with dopamine alone. The firing rate went from 0.37 ± 0.12 to 0.091 ± 0.07 Hz (Fig. 7*C*). The degree of inhibition was similar with or without D1/5R and D2/3R antagonists (Fig. 7*D*).

## Discussion

This report establishes the presence of D4R in GnRH neurons, using RT-PCR and immunocytochemistry, and its role in the dopamine-mediated inhibition of GnRH neurons, using calcium imaging and electrophysiology. Calcium imaging experiments revealed that D4R-mediated inhibition occurs downstream of G_αi/o_ protein. In contrast, activation of D1-like receptors leads to an excitation that requires the G_αs_ protein. Notably, although activation of D1-like or D2-like receptor subfamilies with specific agonists consistently produced either activation or inhibition of GnRH neurons, respectively, the net effect of dopamine was always inhibitory, suggesting mainly activation of D2-like receptors. However, the exact mechanism of the action of dopamine requires caution. Although the canonical pathway of D1-like receptors is excitatory (Pivonello et al., 2007; Podda et al., 2010), the activation of D1-like receptor can be permissive, whereby D1 receptor activation appears necessary for D2 receptor-mediated inhibition (White, 1987; Wachtel et al., 1989). However, in the system used here, dopamine-mediated inhibition persisted in presence of the D1-like receptor antagonist SCH-23390 and the magnitudes of the dopamine inhibition, with or without SCH-23390, were the same suggesting a minimal role of D1-like receptors, if any. Furthermore, as many G-protein-coupled receptors, dopamine receptors can exist as monomers, homodimers and heterodimers and each assembly possesses specific binding and coupling profiles according to occupancy, offering tissue-specific responses (for review, see Maggio et al., 2009; Van Craenenbroeck et al., 2011; Perreault et al., 2014). In our data, the D1-like receptor agonist produced only excitation while dopamine with D2-like receptor antagonist produced both inhibition and excitation, leading to a lack of net effect. The simplest explanation for the latter might be greater binding of dopamine for D2-like receptors and/or a more efficient signaling pathway downstream of these receptors (Schoffelmeer et al., 1994; Skinbjerg et al., 2012). The more convoluted explanation might be that D1-like receptor agonist activates D1-like dimers only, and that dopamine with D2-like receptor antagonist activates both D1-like dimers and D1-like+D2-like dimers via transactivation (Terrillon and Bouvier, 2004).

The complexity of dimerization might rationalize the “apparent” inhibition driven by D1-like receptors in mouse, especially in the cells where the inhibition was equally sensitive to raclopride and SCH-23390 (Liu and Herbison, 2013). In addition, the D2-like receptor mediated inhibition was identified as dependent on activation of D4R and a G_αi/o_ downstream signaling pathway. This observation provides another explanation to the D1-like receptor driven inhibition in mouse. Raclopride is ineffective on D4R (Boy et al., 1998) but SCH-23390 would likely inhibit D1-like+D4R dimers, accounting for the majority of cells reported whose inhibition was sensitive to SCH-23390, but not to raclopride (Liu and Herbison, 2013). Consistent with this, both transcript and protein for D4R were found in GnRH cells. Inhibition of GnRH cells via dopamine D4R is supported by the fact that this receptor, along with the D3R, are downregulated during proestrus (Vastagh et al., 2016), and that it canonically couples to G_αi/o_ protein inwardly rectifying potassium channels (Werner et al., 1996; Inanobe et al., 1999; Wedemeyer et al., 2007), effective inhibitors of GnRH neuronal activity (Klenke et al., 2010; Constantin and Wray, 2016, 2018).

Notably, the D2/3R have been commonly linked to reproductive function. For example, a reduction in the dopaminergic tone and the expression of D2R are observed in women suffering from polycystic ovary syndrome (Chaudhari et al., 2018) and the secretion of luteinizing hormone (LH) is sensitive to D2/3R antagonist in prepubertal females (Lacau de Mengido et al., 1987). However, since discriminative pharmacological tools are recent, the role of the D4R might have been underestimated. For example, D2R+D4R is a functional heteromer that would likely be antagonized by D2/3R antagonist. PCR analysis of cDNA libraries from GnRH neurons support the expression of multiple dopamine receptor subtypes. While the pharmacological data suggest a prominent role of D4R in the control of GnRH neuronal activity by dopamine, the role of the other receptors in the control of GnRH/LH secretion cannot be excluded, especially with dimerization and transactivation mechanisms.

*In vivo*, dopaminergic inputs could regulate GnRH neuronal activity since they have been found around their soma (Jennes et al., 1983; Leranth et al., 1988), i.e., near the site of initiation of APs (Iremonger and Herbison, 2012). In addition, dopamine may regulate GnRH secretion since GnRH neuron nerve terminals also receive dopaminergic inputs (Jennes et al., 1983; Kuljis and Advis, 1989). Dopaminergic receptors have been shown to exhibit a subcellular regionalization (Levey et al., 1993; Hersch et al., 1995). Thus, excitatory D1-like receptors located on GnRH neuron nerve terminals (Fuxe et al., 1988) could be responsible for dopamine-stimulated GnRH secretion (Rasmussen et al., 1986), while D4R located on GnRH cell soma could be responsible for dopamine-inhibited GnRH secretion (Tasaka et al., 1985). Although not exclusively associated with dopaminergic neurotransmission, dopamine-regulated and cAMP-regulated neuronal phosphoprotein, a major convergent point of signaling pathways, has been detected in GnRH nerve terminals (Meister et al., 1988). Finally, indirect effects of dopamine on GnRH secretion cannot be ruled out either (Rotsztejn et al., 1976; Jarjour et al., 1986; James et al., 1987).

In addition to the complexity of GnRH neuron morphology and the dimerization of dopamine receptors, growing evidence supports the physiological role of dopamine receptor heterodimers with other receptors (for review, see Beaulieu et al., 2015). The D4R is structurally a unique receptor, exhibiting a unique third intracellular loop favoring interactions with multiple signaling proteins (Woods, 2010). Notably, exon III which encodes the third loop of the D4R is highly polymorphic (Lichter et al., 1993) and determines signaling efficiency (Rondou et al., 2010). Consequently, D4R is an important player of neuromodulation [α1 and β1 adrenergic receptors (González et al., 2012), glutamate receptor (Price and Pittman, 2001), GABA_A_ receptor (Wang et al., 2002; Azdad et al., 2003; Shin et al., 2003; Graziane et al., 2009; Gasca-Martinez et al., 2010), NMDA receptor (Wang et al., 2003; Andersson et al., 2012), AMPA receptor (Gu et al., 2006; Yuen et al., 2010; Yuen and Yan, 2011)] possibly via Ca^2+^/calmodulin-dependent protein kinase II mechanism (Gu et al., 2006; Yuen et al., 2010; Yuen and Yan, 2011) and/or interfering with receptor trafficking (Graziane et al., 2009; Yuen and Yan, 2009, 2011; Yuen et al., 2010). Consistent with D4R being only a neuromodulator of fertility, mice lacking D4R exhibit behavioral changes (Thanos et al., 2015) but normal fertility (Rubinstein et al., 1997), similar to mice whose kisspeptin neurons exhibit TH deletion (Stephens et al., 2017). Kisspeptin/TH neurons seem to provide a sex-specific pheromonal input to GnRH neurons (Taziaux and Bakker, 2015). Notably, in human, the exon III polymorphism of D4R is linked to the etiology of attention-deficit/hyperactivity disorder (Leung et al., 2017) but has also been linked to normal behaviors such as higher novelty-seeking (Ebstein et al., 1996) and greater sex-specific affective knowledge (Ben-Israel et al., 2015).

Thus, although many nuances remain unclear on the overall action of dopamine and its receptors on reproductive function, our data confirm dopamine as a robust inhibitor of GnRH neuronal activity and pinpoints the D4R as the main integrator of dopamine signal to GnRH neurons, via the activation of a G_αi/o_ protein-dependent signaling pathway.

## Synthesis

Reviewing Editor: Kevin Wickman, University of Minnesota Decisions are customarily a result of the Reviewing Editor and the peer reviewers coming together and discussing their recommendations until a consensus is reached. When revisions are invited, a fact-based synthesis statement explaining their decision and outlining what is needed to prepare a revision will be listed below. The following reviewer(s) agreed to reveal their identity: Naohiro Koshiya, Vance L. Trudeau.

This manuscript describes a comprehensive and well-designed study related to the dopaminergic inhibition of gonadotropin-releasing hormone (GnRH) secreting neurons. The authors bring an array of approaches to the table to show an interesting impact of D4 dopamine receptor activation on GnRH neurons, with potential implications for fertility. The work will set the stage nicely for future studies.

MAJOR CONCERNS - MUST ADDRESS IN REVISION

1) Dopamine easily oxidizes to aminochrome in physiological solutions. How was this reduced or prevented? If it was not, how can one conclude definitively that the effects observed are from dopamine?

2) In a complex sample, immunocytochemisty cannot really prove that a particular protein type is found in a particular cell type (even if RNA transcripts are present) unless that protein is found in an intracellular compartment. Receptors and receptor-mediated responses cannot be assigned to a particular cell type unless the microscopy uses confocal or multiphoton configurations that scan a thin intracellular plane. Related to this concern, the dopaminergic receptor actions measured in this study could occur presynaptically. Without adding data from experiments in which synaptic transmission is blocked, the authors would need to attenuate their conclusions significantly. This may give a clue for the overall perspective with the D4R-D1/5R dissociations.

3) The patch clamp electrophysiology component of this work is relatively incomplete, as compared to the Ca2+ imaging component. If the authors have used both approaches on a single set of preparations and there is agreement across the methodologies, this could be shown before datasets involving Ca2+ imaging alone as a rationale for limiting the investigations to Ca2+ measurements only.

4) While many of the antibodies used in this study seem to be previously characterized, it would help to know that the authors conducted the usual controls (e.g., pre-absorption etc) as part of this study or for previous publications.

5) ​​Line 131: Male mice have hormonal fluctuations, so a better rationale for focusing on male subjects must be provided.

MINOR CONCERNS - SHOULD ADDRESS IN REVISION

1) Line 152-159: Please be consistent with “Delta” vs. “delta” vs. delta symbol. Relatedly, it will be useful to be more specific in the “Delta” designation (e.g., example Delta [Ca+2])

2) Line 167/168, 184/185 and line 305/307). Please incorporate these single sentences into adjacent paragraphs, or build out into stand-alone paragraphs.

3) Line 247-248: The sentence is unclear - what is meant by paradigm in this context?

4) Line 327: this sentence is hard to follow and better explanation is requested. Relatedly, do the authors have any data about potential receptor dimers in their system? This suggestion feels quite speculative.

5) Line 371: recommend replacing “one-of-a-kind” (an unusual phrase for scientific communication) with “unique"

6) Line 381 & 383: recommend avoiding the use of the word “fact”. New data can always change interpretations, so avoiding such phrases is advisable.

7) General issues: overall grammar and syntax can be improved throughout. For example, the authors often drop “the", “a” and others. Some attention to verb tenses will also help the clarity of the report. In addition, some aspects of the figures could be improved. For example, the lines are often too fine and many seem to be distract (e.g., a line joining 2 means to show statistical significance are really necessary; these can be removed). The merged immunofluorescence in Figure 6 could be sharper. The AP plots in Fig 7 are quite small. And, the small red lines on the black AP plots could be very hard to see for some people. Other issues

## Author Response

Synthesis of Reviews:

Synthesis Statement for Author (Required):

This manuscript describes a comprehensive and well-designed study related to the dopaminergic inhibition of gonadotropin-releasing hormone (GnRH) secreting neurons. The authors bring an array of approaches to the table to show an interesting impact of D4 dopamine receptor activation on GnRH neurons, with potential implications for fertility. The work will set the stage nicely for future studies.

We thank the reviewers for their positive words.

MAJOR CONCERNS - MUST ADDRESS IN REVISION

1) Dopamine easily oxidizes to aminochrome in physiological solutions. How was this reduced or prevented? If it was not, how can one conclude definitively that the effects observed are from dopamine?

Dopamine was diluted from stock immediately before experiments as noted in method section. One can only argue that the concentration is lower that what we aimed for due to oxidation. Supporting the specific effect though, DA solutions produce an effect that can be mimicked by various combination of more stable compounds and can be antagonized by DA antagonists.

2) In a complex sample, immunocytochemisty cannot really prove that a particular protein type is found in a particular cell type (even if RNA transcripts are present) unless that protein is found in an intracellular compartment. Receptors and receptor-mediated responses cannot be assigned to a particular cell type unless the microscopy uses confocal or multiphoton configurations that scan a thin intracellular plane. Related to this concern, the dopaminergic receptor actions measured in this study could occur presynaptically. Without adding data from experiments in which synaptic transmission is blocked, the authors would need to attenuate their conclusions significantly. This may give a clue for the overall perspective with the D4R-D1/5R dissociations.

D4R transcripts were found in single cell cDNA. One can always argue about the specificity of an antibody. However, in this report we used 3 different antibodies for D4R. In addition, no primary controls were run for each staining and an example is now included in figure 6. To clarify here, and now added to the manuscript, figure 6A and B show immunofluorescence in a single plan obtained by confocal imaging.

All pharmacological studies in explants/slices were performed while blocking the 2 main synaptic inputs to GnRH neurons, i.e. GABA and glutamate, to ensure a direct effect on GnRH cells. In addition, the conclusion about the role of D4R on GnRH neuronal activity is confirmed using multiple combinations of pharmacological agents.

3) The patch clamp electrophysiology component of this work is relatively incomplete, as compared to the Ca2+ imaging component. If the authors have used both approaches on a single set of preparations and there is agreement across the methodologies, this could be shown before datasets involving Ca2+ imaging alone as a rationale for limiting the investigations to Ca2+ measurements only.

Calcium imaging was thoroughly done in explants due to the cleanliness of this in vitro model (relatively isolated and intact neurons easily manipulated). Only the conclusion of D4R function was challenged in a brain slice because this model comes with issues such as many more cell types and possible interaction, and severed processes which have been shown to influence GnRH activity (Constantin et al., Endocrinology 2012).

4) While many of the antibodies used in this study seem to be previously characterized, it would help to know that the authors conducted the usual controls (e.g., pre-absorption etc) as part of this study or for previous publications.

See. Response #2. We have added that no primary antibody controls were done.

5) Line 131: Male mice have hormonal fluctuations, so a better rationale for focusing on male subjects must be provided.

We were referring to large sex hormone fluctuations (estradiol, progesterone) that occurs through the estrous cycle in females that are known to impact upon GnRH neuron firing. We have clarified this point.

MINOR CONCERNS - SHOULD ADDRESS IN REVISION

1) Line 152-159: Please be consistent with “Delta” vs. “delta” vs. delta symbol. Relatedly, it will be useful to be more specific in the “Delta” designation (e.g., example Delta [Ca+2])

Delta is not a difference in intracellular [Ca2+]i but a difference in frequency between treated period vs previous period. The inconsistencies have been fixed and “delta” was used throughout the manuscript.

2) Line 167/168, 184/185 and line 305/307). Please incorporate these single sentences into adjacent paragraphs, or build out into stand-alone paragraphs.

Done.

3) Line 247-248: The sentence is unclear - what is meant by paradigm in this context?

We have clarified the meaning.

4) Line 327: this sentence is hard to follow and better explanation is requested. Relatedly, do the authors have any data about potential receptor dimers in their system? This suggestion feels quite speculative.

While we show GnRH neurons express more than D4R in our system, here we are discussing the possibility of heterodimers that could explain pharmacological discrepancy.

5) Line 371: recommend replacing “one-of-a-kind” (an unusual phrase for scientific communication) with “unique"

Done

6) Line 381 & 383: recommend avoiding the use of the word “fact”. New data can always change interpretations, so avoiding such phrases is advisable.

Done

7) General issues: overall grammar and syntax can be improved throughout. For example, the authors often drop “the", “a” and others. Some attention to verb tenses will also help the clarity of the report. In addition, some aspects of the figures could be improved. For example, the lines are often too fine and many seem to be distract (e.g., a line joining 2 means to show statistical significance are really necessary; these can be removed). The merged immunofluorescence in Figure 6 could be sharper. The AP plots in Fig 7 are quite small. And, the small red lines on the black AP plots could be very hard to see for some people.

The lines between means were removed. The AP plots are illustrating the quantification of the raw traces on the left. We replaced the red lines on the AP plots with gray bars.
